# Parkinson's disease-associated human ATP13A2 (PARK9) deficiency causes zinc dyshomeostasis and mitochondrial dysfunction

**DOI:** 10.1093/hmg/ddt623

**Published:** 2014-01-07

**Authors:** Jin-Sung Park, Brianada Koentjoro, David Veivers, Alan Mackay-Sim, Carolyn M. Sue

**Affiliations:** 1 Department of Neurogenetics, Kolling Institute of Medical Research, Royal North Shore Hospital and the University of Sydney, St Leonards, New South Wales 2065, Australia and; 2 National Adult Stem Cell Research Centre, Eskitis Institute for Cell and Molecular Therapies, School of Biomolecular and Physical Sciences, Griffith University, Queensland 4111, Australia

## Abstract

Human ATP13A2 (PARK9), a lysosomal type 5 P-type ATPase, has been associated with autosomal recessive early-onset Parkinson's disease (PD). *ATP13A2* encodes a protein that is highly expressed in neurons and is predicted to function as a cation pump, although the substrate specificity remains unclear. Accumulation of zinc and mitochondrial dysfunction are established aetiological factors that contribute to PD; however, their underlying molecular mechanisms are largely unknown. Using patient-derived human olfactory neurosphere cultures, which harbour loss-of-function mutations in both alleles of *ATP13A2*, we identified a low intracellular free zinc ion concentration ([Zn^2+^]_i_), altered expression of zinc transporters and impaired sequestration of Zn^2+^ into autophagy-lysosomal pathway-associated vesicles, indicating that zinc dyshomeostasis occurs in the setting of ATP13A2 deficiency. Pharmacological treatments that increased [Zn^2+^]_i_ also induced the production of reactive oxygen species and aggravation of mitochondrial abnormalities that gave rise to mitochondrial depolarization, fragmentation and cell death due to ATP depletion. The toxic effect of Zn^2+^ was blocked by ATP13A2 overexpression, Zn^2+^ chelation, antioxidant treatment and promotion of mitochondrial fusion. Taken together, these results indicate that human ATP13A2 deficiency results in zinc dyshomeostasis and mitochondrial dysfunction. Our data provide insights into the molecular mechanisms of zinc dyshomeostasis in PD and its contribution to mitochondrial dysfunction with ATP13A2 as a molecular link between the two distinctive aetiological factors of PD.

## INTRODUCTION

Parkinson's disease (PD) is the most common movement disorder, typically identified with clinical manifestations of tremor, bradykinesia, rigidity and postural instability. Degeneration of dopaminergic neurons in the substantia nigra pars compacta (SNpc) and formation of intracellular inclusion bodies (Lewy bodies) serve as histopathological hallmarks of PD. More than 90% of patients present as sporadic cases where the cause of the disease is unknown (sporadic PD), whereas ∼10% of PD patients have identifiable monogenic causes (familial PD). To date, 18 genes or loci in the human genome have been associated with familial PD (1).

The *ATP13A2* gene (*PARK9*, MIM# 610513) encodes a lysosomal type 5 P-type ATPase. Mutations in *ATP13A2* have been associated with an autosomal recessive levodopa-responsive early-onset parkinsonism, known as Kufor–Rakeb syndrome (KRS, MIM# 606693) (2). KRS patients present with typical PD manifestations alongside other clinical features such as supranuclear gaze palsy, facial-faucial myoclonus and spasticity (3). Mutations identified in most KRS patients follow an autosomal recessive trait involving two mutant alleles (homozygotes or compound heterozygotes) that cause mRNA degradation, protein misfolding/truncation and degradation (2–5). ATP13A2 protein has been localized to several cellular acidic vesicles, including lysosomes and autophagosomes (2–10). It was therefore proposed that ATP13A2 functions in the autophagy-lysosomal pathway (ALP). In support of this, mutations in ATP13A2 have been associated with neuronal ceroid lipofuscinosis, a lysosomal storage disorder, in humans and dogs (11–13) and lysosomal dysfunction in KRS-patient-derived cell models (8, 14). ATP13A2 has also been predicted to be a cation pump, based on its structural similarity to other proteins in the type 5 P-type ATPase family. Several metal ions have been reported as potential substrates (15). Among them, ionic manganese (Mn^2+^) has been the cation subject of the most extensive investigation, because it is also a known environmental risk factor for PD. Several groups have demonstrated an exaggerated Mn^2+^ toxicity at high doses in *ATP13A2*-silenced yeast and mammalian cell models (9, 10, 16). In these models, overexpression of wild-type, but not mutant ATP13A2, conferred protection against Mn^2+^ toxicity. Despite the apparent interaction of Mn^2+^ in disease models, the cationic selectivity of endogenous human ATP13A2 for other metal ions remains to be determined.

In addition to manganese, zinc has been shown to interact with peptide fragments of ATP13A2 (17). Zinc, which is enriched in the brain, is an essential biometal required in numerous biological processes to maintain normal cell function. The intracellular concentration of biologically active free zinc ions (Zn^2+^) is tightly regulated by zinc transporters to a diminutive level due to their potential toxicity, whereas the majority of intracellular Zn^2+^ exists in an inactive form either bound to zinc-binding proteins (i.e. metallothioneins) or sequestered in cellular organelles (18). Zinc dyshomeostasis has been linked with several neurodegenerative diseases including PD. Elevated levels of zinc have been found in the SNpc and other tissues of PD patients (19–21), and zinc has been identified as an environmental risk factor for PD (22). Despite the potential importance of zinc in the pathogenesis of PD, its aetiological role remains largely unknown.

Excessive Zn^2+^ levels are also known to impair cellular energy production through an inhibitory action on mitochondria (23). Mitochondria generate the majority of cellular energy in the form of ATP via oxidative phosphorylation and produce detrimental reactive oxygen species (ROS) as a byproduct of this process. Mitochondrial dysfunction was initially linked to the pathogenesis of PD when 1-methyl-4-phenyl-1,2,3,4-tetrahydropyridine (MPTP), a potent mitochondrial complex I inhibitor and a neurotoxic contaminant in the synthetic recreational opioid desmethylprodine, was linked to dopaminergic cell death in the SNpc, resulting in a PD-like syndrome (24). Since then, mitochondrial dysfunction has been recognized as a major contributor to the aetiology of sporadic (25, 26) and familial PD (27–30). A recent discovery that zinc accumulation contributes to and conversely, zinc chelation protects against MPTP-induced PD has highlighted a link between zinc and mitochondrial function in the pathogenesis of PD (31).

We previously reported that pathogenic compound heterozygous mutations in *ATP13A2* caused loss of ATP13A2 expression and mitochondrial dysfunction (3, 28). In this study, we have identified zinc dyshomeostasis in our human olfactory neurosphere (hONs) disease model system (32). The patient-derived hONs cells displayed a lower intracellular free zinc ion concentration ([Zn^2+^]_i_) with a decreased capacity to sequester Zn^2+^ into the ALP vesicles and altered expression of zinc transporters. Pharmacological treatments that elevated the [Zn^2+^]_i_ were found to exacerbate the loss of mitochondrial function, leading to mitochondrial fragmentation and cell death as a result of ATP depletion. These findings indicate that loss of human ATP13A2 causes zinc dyshomeostasis and abnormal energy metabolism, providing evidence that ATP13A2 is a molecular link between abnormal zinc metabolism and mitochondrial dysfunction in the pathogenesis of PD.

## RESULTS

### ATP13A2^−/−^ hONs cells are vulnerable to elevated [Zn^2+^]_i_

**Figure 1.**
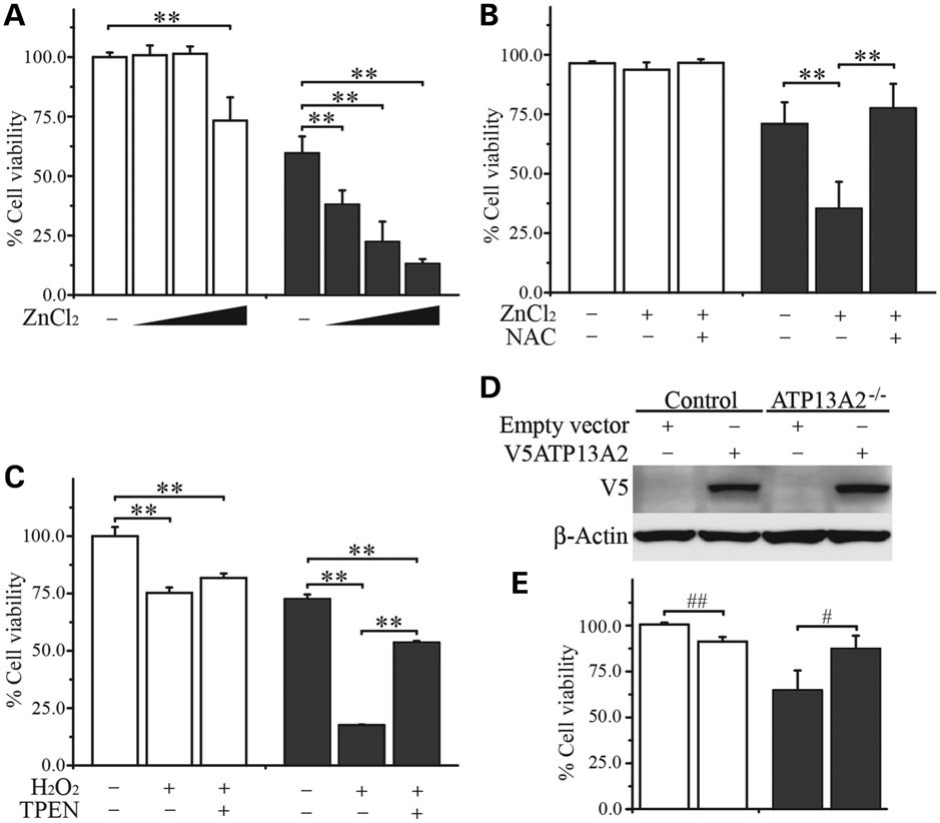
Zn^2+^-induced ROS-mediated cell death in ATP13A2^−/−^ hONs cells. hONs cells were tested for zinc sensitivity using the Neutral red uptake assay. (**A**) Increasing doses of ZnCl_2_ (0, 100, 112.5 and 125 µm) significantly reduced the cell viability of ATP13A2^−/−^ cells (grey bar) in a dose-dependent manner, while the same treatment induced a significant change only at the highest dose in the control cells (white bar). (**B**) The cytotoxic effect of ZnCl_2_ (112.5 µm) on ATP13A2^−/−^ cells was completely reversed by co-treatment with NAC (1 mm), an antioxidant agent. (**C**) H_2_O_2_ (1 mm) reduced cell viability in both hONs cell lines, but to a greater extent in ATP13A2^−/−^ cells compared with the control. The H_2_O_2_-mediated toxicity was significantly reduced by TPEN (1 µm), a Zn^2+^ chelator. (**D**) Western blot analysis detected the expression of V5-tagged ATP13A2 (V5ATP13A2) in the hONs cells transduced with V5ATP13A2 expressing lentivirus, but not in the cells transduced with lentivirus carrying an empty vector. β-Actin was used as a loading control. (**E**) ATP13A2 overexpression protected Zn^2+^-mediated cytotoxicity induced by 100 µm ZnCl_2_ in ATP13A2^−/−^ cells compared with the empty vector control. Values in the graphs are represented as mean ± SD. NAC; *N*-acetyl-cysteine, TPEN, *N*,*N*,*N*′,*N*′-tetrakis(2-pyridylmethyl)ethylenediamine. #*P* < 0.05 and ##*P* < 0.01 by Mann–Whitney *U* test and **P < 0.01 by Kruskal–Wallis one-way ANOVA followed by *post hoc* Tukey's HSD multiple comparison test.

In order to determine the effect of excessive zinc levels in the setting of ATP13A2 deficiency, we exposed hONs cells with compound heterozygous loss-of-function mutations (c.3253delC and c.3176T>G) in *ATP13A2* (3), to increasing doses of ZnCl_2_ and measured the cell viability using the Neutral red uptake assay (33). hONs with ATP13A2 deficiency are denoted as ATP13A2^−/−^ hereafter. In the vehicle-treated groups, ATP13A2^−/−^ cells consistently showed a 20–40% lower retention of Neutral red compared with the control (Fig. 1). Neutral red is a weakly cationic dye and retained in the lysosomes depending on their pH (33) and the lower retention of Neutral red detected under vehicle treatment reflected a higher lysosomal pH in ATP13A2^−/−^ KRS-patient cells (8, 14). When treated with ZnCl_2_, ATP13A2^−/−^ cells showed a dose-dependent and significant decrease in cell viability (*P* < 0.01), whereas the control cells demonstrated cytotoxicity only at the highest dose tested (*P* < 0.01, Fig. 1A). As Zn^2+^ has been shown to increase mitochondrial ROS production (34), we then examined whether ROS was involved in the observed Zn^2+^-induced cytotoxicity. The Zn^2+^-induced reduction of cell viability in ATP13A2^−/−^ cells was completely reversed by the introduction of an antioxidant, *N*-acetyl-cysteine (NAC), indicating that Zn^2+^ toxicity is elicited by increased ROS production in ATP13A2^−/−^ cells (Fig. 1B). Hydrogen peroxide (H_2_O_2_), an ROS known to increase [Zn^2+^]_i_ by inducing the release of Zn^2+^ from zinc-binding proteins (31), significantly reduced cell viability, to a greater extent in ATP13A2^−/−^ cells (*P* < 0.01, Fig. 1C). Furthermore, the specific Zn^2+^ chelator, *N*,*N*,*N*′,*N*′-tetrakis(2-pyridylmethyl) ethylenediamine (TPEN), protected against H_2_O_2_-mediated cytotoxicity, strongly supporting the involvement of Zn^2+^ in H_2_O_2_-mediated cytotoxicity. Next, we overexpressed wild-type ATP13A2 in ATP13A2^−/−^ cells and treated ZnCl_2_ to test whether restoration of ATP13A2 expression reverses Zn^2+^ cytotoxicity. Western blot analysis confirmed expression of V5-tagged wild-type ATP13A2 (V5ATP13A2) in both control and ATP13A2^−/−^ cells after lentivirus transduction (Fig. 1D). V5ATP13A2 expression significantly protected Zn^2+^-mediated cytotoxicity in ATP13A2^−/−^ cells (Fig. 1E), whereas a similar overexpression of V5ATP13A2 was slightly toxic to the control cells as previously reported (6). Cytotoxicity/cell viability measured by the lactate dehydrogenase activity in the culture media of hONs cells and the Trypan blue exclusion assay was consistent with the results of the Neutral red uptake assay (Supplementary Material, Fig. S1A-G), confirming the increased cytotoxicity of Zn^2+^ in ATP13A2^−/−^ cells. Together, these findings support the existence of zinc dyshomeostasis in ATP13A2^−/−^ cells, conferring sensitivity to treatments that induce an increase in [Zn^2+^]_i_ and ROS as an effector of Zn^2+^-mediated toxicity.

### [Zn^2+^]_i_ is lower in ATP13A2^−/−^ hONs cells

**Figure 2.**
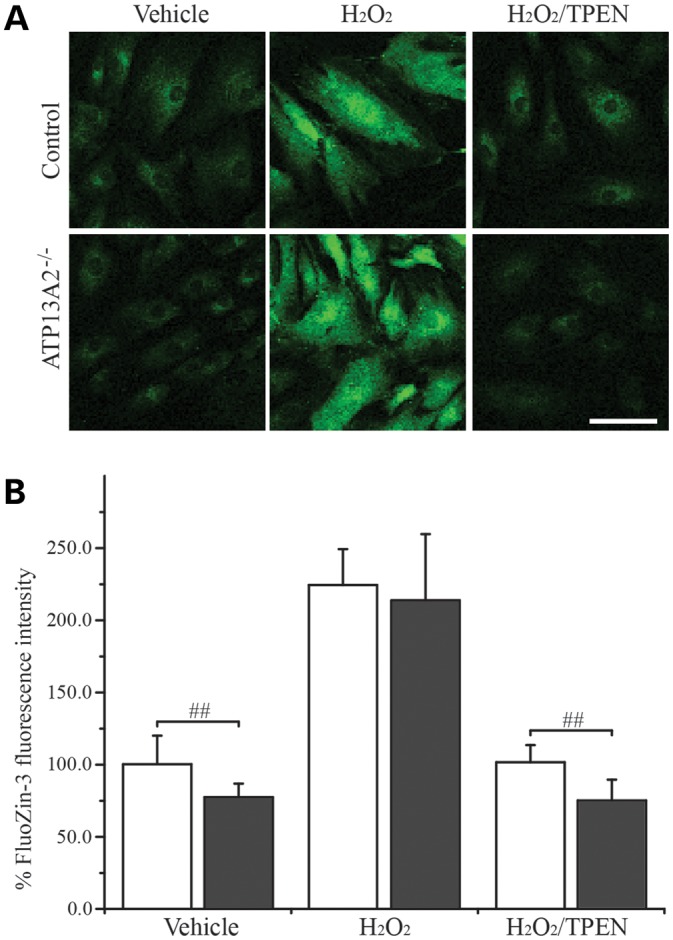
Reduced [Zn^2+^]_i_ in ATP13A2^−/−^ hONs cells. [Zn^2+^]_i_ was determined by quantification of the FluoZin-3 fluorescence in hONs cells. Cell images used for analysis were acquired at three to four randomly selected locations on a coverslip (a total of 3-4 coverslips per group in two independent experiments). There was no difference in the number of cells per coverslip between the cell lines (186.9 ± 46.3 for the control and 201.0 ± 38.4 for ATP13A2^−/−^ cells, *P* = 0.15 in a two-tailed Student's *t*-test). (**A**) Representative confocal images are presented for control (upper panel) and ATP13A2^−/−^ (bottom panel) cells treated as indicated. Scale bar = 100 µm. (**B**) Quantification of fluorescence from the cells revealed a significantly reduced FluoZin-3 signal for ATP13A2^−/−^ cells (grey bar) compared with the control (white bar) under vehicle treatment. H_2_O_2_ treatment increased FluoZin-3 signals to a similar extent in both the cell lines, while TPEN treatment reversed the H_2_O_2_-induced increase in FluoZin-3 signals to basal levels. Values in the graphs are represented as mean ± SD. TPEN, *N,N,N*′,*N′*-tetrakis(2-pyridylmethyl)ethylenediamine. ##; *P* < 0.01 by Mann–Whitney *U* test.

Excessive Zn^2+^ concentration is known to be detrimental to cellular function (23, 35), necessitating the maintenance of low [Zn^2+^]_i_. As our cytotoxicity tests suggested that zinc homeostasis was disturbed in ATP13A2^−/−^ cells, we assessed [Zn^2+^]_i_ using FluoZin-3 (Fig. 2). FluoZin-3 is a Zn^2+^ specific dye that exhibits green fluorescence upon binding to Zn^2+^ and has been widely used to measure [Zn^2+^]_i_ (31, 34, 36, 37). In the vehicle-treated groups, ATP13A2^−/−^ cells showed an average of 23% reduction in the FluoZin-3 intensity compared with the control (*P* < 0.01), indicating lower [Zn^2+^]_i_ in ATP13A2^−/−^ cells. Upon exposure to H_2_O_2_, both hONs cell lines showed a >2-fold increase in the FluoZin-3 fluorescence intensity, which was not significantly different between the two cell lines (*P* = 0.51). H_2_O_2_-induced release of Zn^2+^ was efficiently reverted to basal levels by co-treatment with TPEN, confirming the specificity of Zn^2+^ in the H_2_O_2_-induced increase of FluoZin-3 fluorescence intensity. The lower [Zn^2+^]_i_ in ATP13A2^−/−^ cells was also confirmed using another Zn^2+^-specific fluorescent dye, Zinpyr-1, by flow cytometry (Supplementary Material, Fig. S2).

### Altered expression of zinc transporters in ATP13A2^−/−^ hONs cells

**Figure 3.**
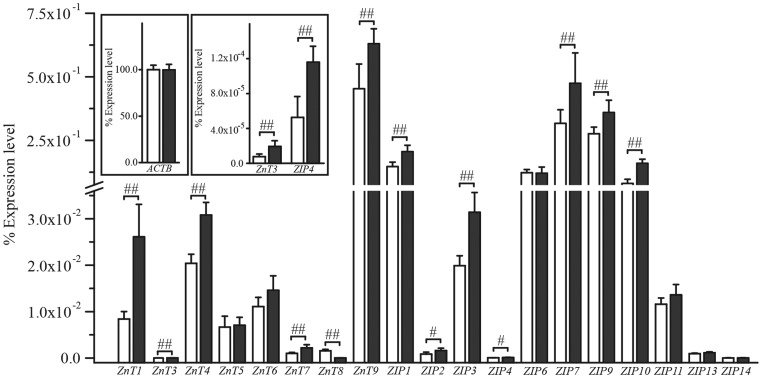
Altered expression of ZnTs/ZIPs in ATP13A2^−/−^ cells. cDNA from hONs cells was analysed to determine the mRNA expression level of ZnTs/ZIPs using a quantitative real-time RT–PCR. White bars in the graphs represent the control and grey bars represent ATP13A2^−/−^ cells. Among the genes investigated, 19 genes (8 *ZnTs* and 11 *ZIPs*) were expressed in the hONs cells, while the expression of *ZnT2*, *ZIP5*, *ZIP8* and *ZIP12* mRNA was not detected with the PCR conditions used (see Materials and Methods). There was no difference detected in the expression of *β-actin* (*ACTB*) encoding between the two cell lines (inset in the left of the top corner).The expression levels for six *ZnTs* (*ZnT1*, *ZnT3*, *ZnT4*, *ZnT7*, *ZnT8* and *ZnT9)* and seven *ZIPs* (*ZIP1*, *ZIP2*, *ZIP3*, *ZIP4*, *ZIP7*, *ZIP9* and *ZIP10*) were significantly upregulated in ATP13A2^−/−^ cells, while *ZnT8* mRNA expression was significantly downregulated. ZnT, zinc transporters encoded by *solute carrier family 30* genes (*SLC30A1* to *A9*); ZIP, ZRT/IRT-related proteins encoded by *solute carrier family 39* genes (*SLC39A1* to *A14*). All reactions were repeated twice in triplicate. Values in the graphs are represented as mean ± SD. #*P* < 0.05 and ##*P* < 0.01 by Mann–Whitney *U* test.

To further assess the impact of ATP13A2 deficiency on zinc homeostasis, we evaluated changes in the expression levels of zinc transporters. To maintain zinc homeostasis, zinc transporters that are located in the membrane of various cellular organelles act to pump Zn^2+^ across the membrane, playing a crucial role in modulating [Zn^2+^]_i_ (35). There are two distinct gene families involved in Zn^2+^ transportation; 9 *solute carrier family 30* genes encode zinc transporters (ZnTs) that mediate efflux of Zn^2+^ (decreasing cytosolic Zn^2+^), and ZRT/IRT-related proteins (zinc importing proteins, ZIP) encoded by 14 *solute carrier family 39* genes that facilitate influx of Zn^2+^ (increasing cytosolic Zn^2+^ levels). We examined the gene expression of all *ZnT* and *ZIP* genes and *ACTB* encoding β-actin as a housekeeping gene in hONs cells using a quantitative real-time RT–PCR (qRT–PCR) (Fig. 3). Among the genes investigated, 19 (8 *ZnTs* and 11 *ZIPs*) were expressed in the hONs cells, while the expression of *ZnT2*, *ZIP5*, *ZIP8* and *ZIP12* was not detected with the PCR conditions employed (see Materials and Methods). There was no difference in the expression of *ACTB*. We found alterations in the expression levels of the majority of zinc pumps (13 out of 19 genes; 6 *ZnTs* and 7 *ZIPs*) in ATP13A2^−/−^ cells compared with the control, suggesting altered Zn^2+^ dynamics through the expression of zinc pumps: *ZnT1*, *ZnT3∼4*, *ZnT7∼9*, *ZIP1∼4*, *ZIP7* and *ZIP9∼10*. All but one (*ZnT8*) of the ZnT/ZIP transcripts were upregulated in ATP13A2^−/−^ cells. These findings, together with the observed lower [Zn^2+^]_i_, are indicative of zinc dyshomeostasis in the presence of ATP13A2 deficiency.

### Impaired sequestration of Zn^2+^ into the ALP vesicles in ATP13A2^−/−^ hONs cells

**Figure 4.**
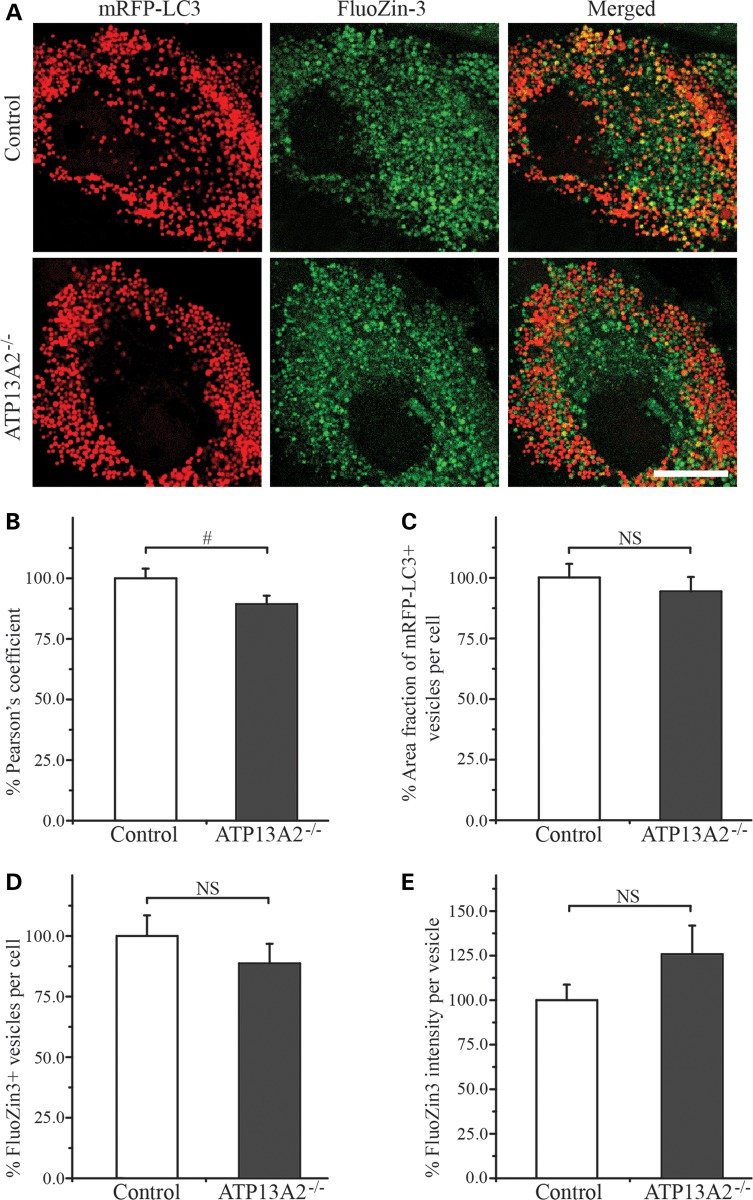
Reduction of Zn^2+^ levels in the ALP vesicles in ATP13A2^−/−^ cells. hONs cells expressing mRFP-LC3 were stained with FluoZin-3 after induction of accumulation of the mRFP-LC3-positive vesicles and release of Zn^2+^ from zinc-bound proteins (see Materials and Methods). (**A**) Representative confocal images are presented for the control (upper panels) and ATP13A2^−/−^ cells (bottom panels). Merged images (right panels) of mRFP-LC3 (red, left panel) and FluoZin-3 (green, middle panel) show yellow puncta, indicating co-localization of ALP vesicles with increased Zn^2+^. Scale bar = 20 µm. (**B**) Pearson's coefficient for co-localization between mRFP-LC3 and FluoZin-3 signals was significantly decreased in ATP13A2^−/−^ cells (grey bar) compared with the control (white bar) (*n* = 47, 11–13 cells per coverslip from four coverslips in two independent experiments). The area fraction occupied by mRFP-LC3-positive vesicles per cell (**C**), the number of FluoZin-3-positive vesicles per cell (**D**) and the FluoZin-3 intensity per vesicle (**E**) were not significantly different between the control and ATP13A2^−/−^ cells. Values in the graphs are represented as mean ± SEM. NS, not significant; #*P* < 0.05 by Mann–Whitney *U* test.

ATP13A2 localizes to intracellular acidic vesicles, including autophagosomes, early/late endosomes and lysosomes (2–10). Based on the reported location of ATP13A2 and the observed zinc dyshomeostasis in our ATP13A2^−/−^ cells, we hypothesized that ATP13A2 is majorly involved in transporting Zn^2+^ across the membrane of ALP vesicles and loss of ATP13A2 impairs the capacity to transport Zn^2+^ into these vesicles. To test the hypothesis, we generated hONs cells expressing mRFP-LC3 to visualize LC3-positive ALP vesicles, including autophagolysosomes (38), and stained them with FluoZin-3 under the induction of accumulation of the ALP vesicles and increase in [Zn^2+^]_i_ (see Materials and Methods for details). The control cells displayed a higher number of vesicles positive for both mRFP-LC3 and FluoZin-3, when compared with ATP13A2^−/−^ cells (Fig. 4A). Further analysis revealed that the Pearson's co-localization coefficient was significantly reduced in ATP13A2^−/−^ cells compared with the control (*n* = 47, *P* < 0.05), indicating a lower number of mRFP-LC3-positive vesicles containing Zn^2+^ in ATP13A2^−/−^ cells (Fig. 4B). The area occupied by mRFP-LC3-positive vesicles per cell did not differ between the two cell lines (*P* = 0.44, Fig. 4C), negating the possibility of random detection of the decreased co-localization in the ATP13A2^−/−^ cells. In addition, there was no difference in the number of FluoZin-3-positive vesicles per cell (*P* = 0.33, Fig. 4D) or the FluoZin-3 intensity per vesicle (*P* = 0.29, Fig. 4E) between the two cell lines. These results indicate that the sequestration of Zn^2+^ into ALP vesicles is impaired by the loss of ATP13A2.

### ATP13A2^−/−^ hONs cells have impaired mitochondrial function

**Figure 5.**
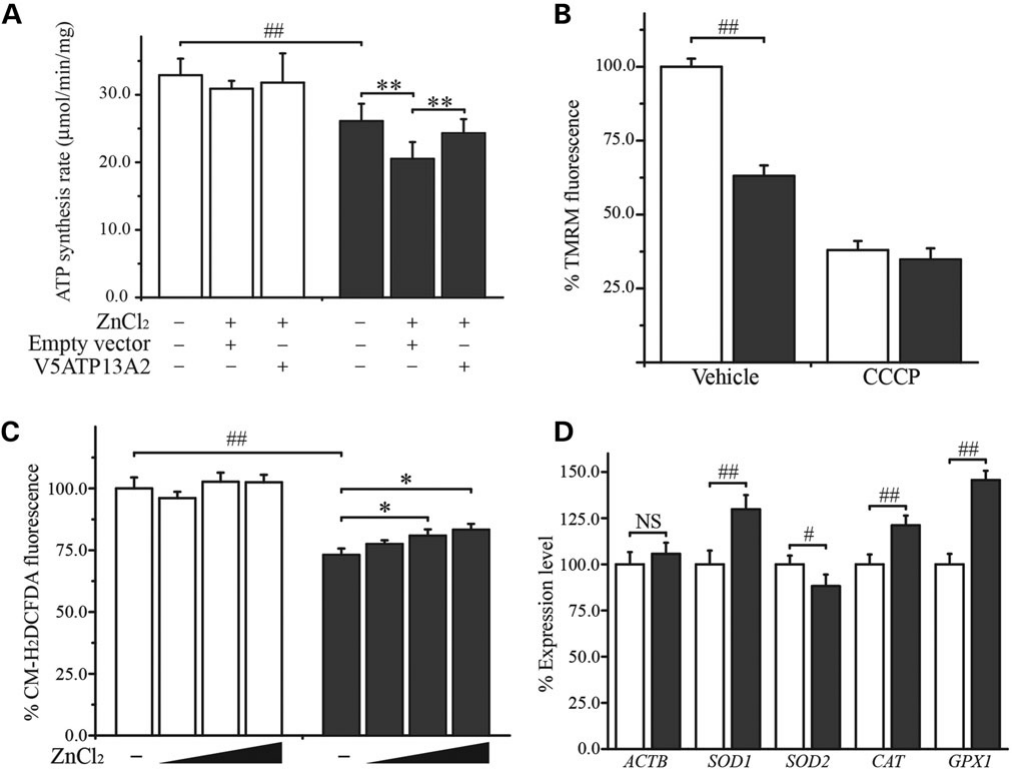
Mitochondrial dysfunction and Zn^2+^-mediated ROS production in ATP13A2^−/−^ cells. Mitochondrial function and ROS production were assessed in hONs cells exposed to ZnCl_2._ (**A**) ATP production rate was significantly lower in ATP13A2^−/−^ cells (grey bars) compared with the control (white bars) under basal conditions. Upon exposure to 100 µm ZnCl_2_, ATP production rate was significantly reduced in the ATP13A2^−/−^ cells transduced with lentivirus carrying an empty vector, which was completely reversed by overexpression of V5-tagged wild-type ATP13A2 (V5ATP13A2). (**B**) TMRM labelling was significantly reduced in ATP13A2^−/−^ cells compared with the control. Treatment of the cells with the mitochondrial uncoupler CCCP decreased TMRM labelling to a similar extent in both the cell lines. (**C**) CM-H_2_DCFDA was used to detect H_2_O_2_ in hONs cells. In the vehicle-treated groups, ATP13A2^−/−^ cells displayed a significantly lower CM-H_2_DCFDA signal compared with the control. When treated with increasing concentrations of ZnCl_2_ (0, 100, 500, 1000 µm) for 30 min, ATP13A2^−/−^ cells displayed a dose-dependent increase in CM-H_2_DCFDA fluorescence signals with a significant increase at concentrations >500 µm. (**D**) Quantitative real-time RT–PCR detected a significant upregulation in the expression level of genes encoding the cellular antioxidant enzymes; superoxide dismutase 1 (*SOD1*), catalase (*CAT*) and glutathione peroxidase 1 (*GPX1*) and a significant down-regulation in superoxide dismutase 2 (*SOD2*), while *β-actin* mRNA (*ACTB*) was expressed at similar levels. All reactions were repeated twice in triplicate. Values in the graphs are represented as mean ± SD. CCCP, carbonyl cyanide 3-chlorophenylhydrazone. #*P* < 0.05 and ##*P* < 0.01 by Mann–Whitney *U* test and *P < 0.05 and **P < 0.01 by Kruskal–Wallis one-way ANOVA followed by *post hoc* Tukey's HSD multiple comparison test.

We and others have reported impaired mitochondrial function in fibroblasts from KRS patients (28) and *ATP13A2*-silenced cell models (6, 39). We therefore assessed mitochondrial function in our hONs cells. The cellular ATP production rate was significantly lower in the ATP13A2^−/−^ cells when compared with controls (32.9 ± 2.4 for control and 26.1 ± 2.6 for ATP13A2^−/−^ cells, *P* < 0.01, Fig. 5A). Upon exposure to ZnCl_2_, ATP13A2^−/−^ cells showed a significant reduction in ATP production rate (*P* < 0.05), which was completely blocked by V5ATP13A2 overexpression (*P* < 0.01 compared with the ZnCl_2_-treated empty vector control), while the same treatments did not change ATP production rate in the control cells. ATP13A2^−/−^ cells showed an average of 37% reduction in tetramethylrhodamine methyl ester perchlorate (TMRM) labelling compared with the control under vehicle treatment (*P* < 0.01), indicative of a lower mitochondrial membrane potential (Δ*Ψ*_m_, Fig. 5B). Notably, there was no difference in total mitochondrial mass between the cell lines when measured using the mitochondria-specific dye, MitoTracker Green (Supplementary Material, Fig. S3). When cells were treated with carbonyl cyanide 3-chlorophenylhydrazone (CCCP), a Δ*Ψ*_m_ uncoupling agent, TMRM retention was reduced to a similar degree in both the cell lines (*P* = 0.75).

### Zn^2+^-mediated ROS production and altered expression of antioxidant genes in ATP13A2^−/−^ hONs cells

Zn^2+^ accumulates in mitochondria via Zn^2+^ transporting uniporters, with a resultant increase in ROS production (34, 36). Given that ROS was found to be an effector of Zn^2+^-mediated cytotoxicity in ATP13A2^−/−^ cells (Fig. 1), we exposed hONs cells to ZnCl_2_ to examine whether exogenous Zn^2+^ induced ROS production in hONs cells. ROS levels were assessed using fluorescent indicators specific for superoxide (O_2_^−^) production (MitoSox Red) and H_2_O_2_ production (CM-H_2_DCFDA). Surprisingly, the basal H_2_O_2_ production was lower by an average of 27% in ATP13A2^−/−^ cells compared with the control (*P* < 0.01, Fig. 5C). However, when exposed to high concentrations of ZnCl_2_, H_2_O_2_ production was rapidly (<30 min) induced in ATP13A2^−/−^ cells while only minimal changes were detected in the control. Although ZnCl_2_ treatment induced O_2_^−^ production in hONs cells, the levels of O_2_^−^ were comparable between the cell lines under both vehicle and ZnCl_2_ treatment conditions (data not shown). Notably, the maximum dose of ZnCl_2_ used to induce ROS production did not cause any appreciable cell death under the given exposure conditions (Supplementary Material, Fig. S4). To determine whether the cause of reduced ROS production in ATP13A2^−/−^ cells resulted from a compensatory activation of the antioxidant enzyme systems, we examined the expression levels of the genes encoding antioxidant enzymes using qRT–PCR. All the genes examined were expressed at variable mRNA levels in ATP13A2^−/−^ cells when compared with the control, while there was no difference detected in the expression of *ACTB* (Fig. 5D); a significant elevation was detected for superoxide dismutase 1 (*SOD1*, 129.8% ± 7.7, *P* < 0.01), catalase (*CAT*, 121.1% ± 5.3, *P* < 0.01) and glutathione peroxidase 1 (*GPX1*, 145.6% ± 5.1, *P* < 0.01), while the level of superoxide dismutase 2 transcripts (*SOD2*, 88.2% ± 6.3, *P* < 0.05) was decreased. These findings confirm the involvement of ROS in Zn^2+^-mediated cytotoxicity in ATP13A2^−/−^ cells and also suggest that a loss of ATP13A2 results in altered ROS metabolism, which contributes to an increased susceptibility to Zn^2+^ and induction of protective changes in the cellular antioxidant system.

### Zn^2+^ disrupts Δ*Ψ*_m_ in ATP13A2^−/−^ hONs cells

**Figure 6.**
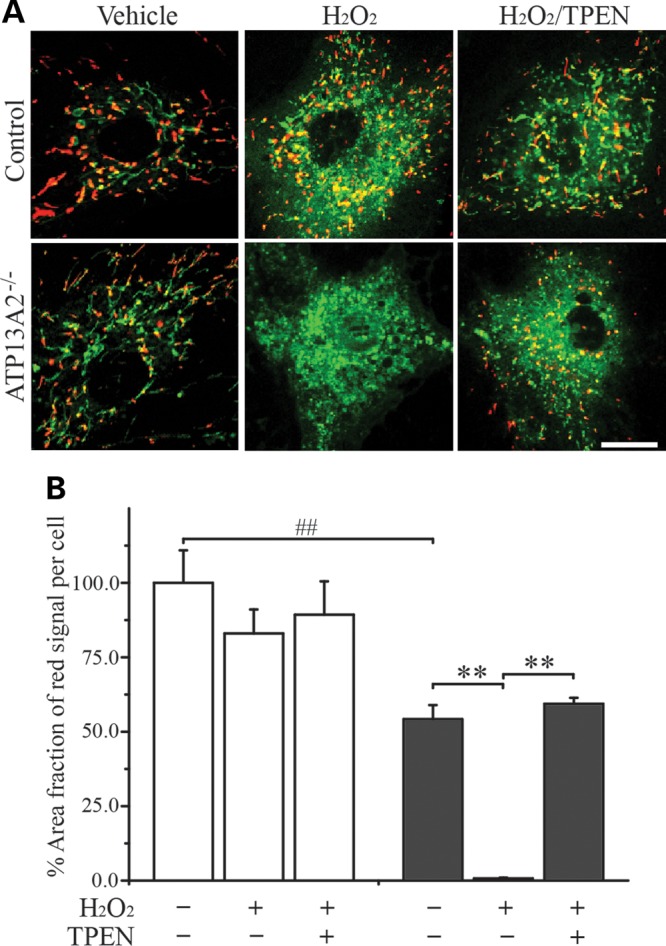
Detrimental effect of elevated [Zn^2+^]_i_ on Δ*Ψ*_m_ in ATP13A2^−/−^ hONs cells. hONs cells were treated with H_2_O_2_ to increase [Zn^2+^]_i_ and stained with 5,5′,6,6′-tetrachloro-1,1′,3,3′-tetraethylbenzimidazolylcarbocyanine iodide (JC-1) to examine the effect of elevated Zn^2+^ levels on mitochondrial membrane potential (Δ*Ψ*_m_). (**A**) Representative confocal images are presented for the control (upper panels) and ATP13A2^−/−^ cells (bottom panels) treated as indicated. Red fluorescence indicates mitochondria with normal Δ*Ψ*_m_ and green fluorescence mitochondria with low Δ*Ψ*_m_ (e.g. damaged mitochondria). The increase of green fluorescence detected in the H_2_O_2_-treated groups is due to cytoplasmic diffusion of JC-1 monomers. Scale bar = 20 µm. (**B**) The area occupied by red fluorescing mitochondria in the vehicle-treated groups was significantly lower in ATP13A2^−/−^ cells (grey bar) compared with the control (white bar). H_2_O_2_ (0.95 mm) treatment significantly decreased the red signal in ATP13A2^−/−^ cells compared with the vehicle treatment, while co-treatment of H_2_O_2_ and TPEN (1 µm) reversed the toxic effect of H_2_O_2_ alone on Δ*Ψ*_m_ (*n* = 24–28, 11–16 cells per coverslip were analysed in two independent experiments). Values in the graphs are represented as mean ± SEM. TPEN, *N*,*N*,*N*′,*N*′-tetrakis(2-pyridylmethyl)ethylenediamine. ##*P* < 0.01 by Mann–Whitney *U* test and **P < 0.01 by Kruskal–Wallis one-way ANOVA followed by *post hoc* Tukey's HSD multiple comparison test.

To assess the effect of [Zn^2+^]_i_ on ΔΨ_m_, we exposed hONs cells to H_2_O_2_ and examined changes in 5,5′,6,6′-tetrachloro-1,1′,3,3′-tetraethylbenzimidazolylcarbocyanine iodide (JC-1) fluorescence. JC-1 is a cationic dye that has been utilized to monitor Δ*Ψ*_m_ through its capacity to exhibit green fluorescence when in a monomeric form in the cytoplasm or in mitochondria with low Δ*Ψ*_m_ (e.g. damaged mitochondria) and red fluorescence upon formation of J-aggregates in mitochondria with normal-to-high Δ*Ψ*_m_ (e.g. healthy mitochondria). Under vehicle treatment, ATP13A2^−/−^ cells displayed on average 46% lower proportion of red mitochondria compared with the control (*P* < 0.01, Fig. 6), consistent with the result of the TMRM assay. Exposure to H_2_O_2_ reduced the area fraction of red mitochondria in both cell lines, but to a significant extent in ATP13A2^−/−^ cells (*P* < 0.01). The toxic effect of H_2_O_2_ on Δ*Ψ*_m_ in ATP13A2^−/−^ cells was blocked by co-treatment with TPEN (*P* < 0.01), confirming the involvement of Zn^2+^ in the H_2_O_2_-mediated reduction of Δ*Ψ*_m_. The noticeable increase in green fluorescence observed with H_2_O_2_ treatment is due to cytoplasmic diffusion of JC-1 monomers.

### Zn^2+^ induces mitochondrial fragmentation, loss of mitochondrial function and cell death in ATP13A2^−/−^ hONs cells

**Figure 7.**
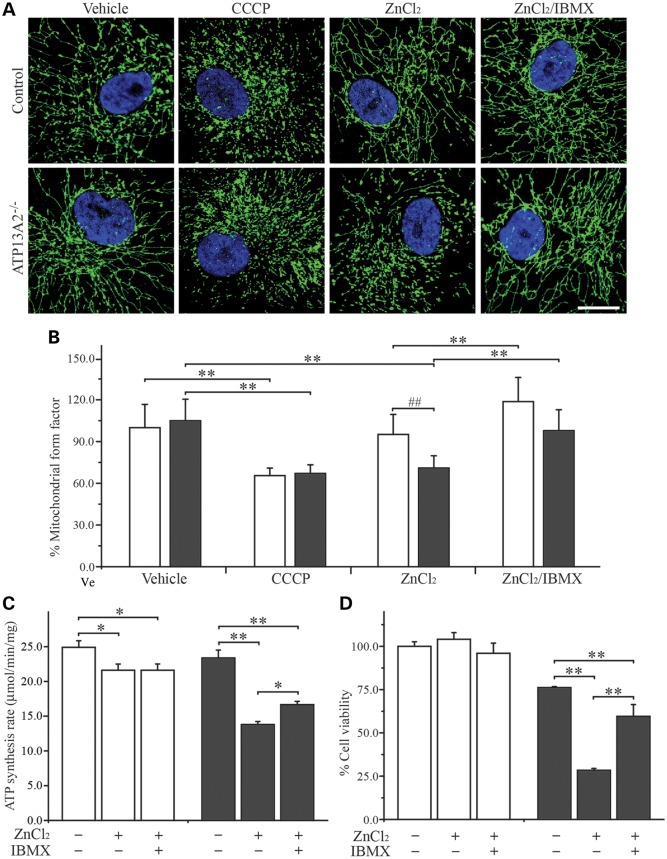
Zn^2+^-mediated mitochondrial fragmentation in ATP13A2^−/−^ hONs cells. hONs cells were treated with either ZnCl_2_ alone or ZnCl_2_ with IBMX and assessed for mitochondrial interconnectivity, ATP production rate and cell viability. (**A**) Cells were immunologically stained for Grp75 (green), a mitochondrial matrix protein and the nuclei were stained with 4',6-diamidino-2-phenylindole (blue). Mitochondrial form factor was calculated to determine the degree of mitochondrial interconnectivity (see Materials and Methods). Representative confocal images are presented for the control (upper panels) and ATP13A2^−/−^ cells (bottom panels) that were treated as indicated. Scale bar = 20 µm. (**B**) The mitochondrial form factor was found to be comparable between the control (white bar) and ATP13A2^−/−^ (grey bar) cells in the vehicle control groups, while CCCP treatment reduced the mitochondrial form factor significantly in both cell lines, indicating mitochondrial fragmentation (*n* = 65, 15–18 cells per coverslip from four coverslips in two independent experiments). Conversely, ZnCl_2_ treatment decreased the mitochondrial form factor in ATP13A2^−/−^ cells, while there was only mild reduction detected in the control. Promotion of mitochondrial fusion using IBMX treatment, prevented ZnCl_2_-mediated mitochondrial fragmentation in ATP13A2^−/−^ cells and further increased mitochondrial interconnectivity in the control. (**C**) ZnCl_2_ (100 µm) treatment caused a significant reduction in ATP production rate in both the cell lines, although to a greater extent in ATP13A2^−/−^ cells (grey bars) compared with the control (white bars). IBMX co-treatment significantly blocked the Zn^2+^-mediated reduction in the ATP production rate in ATP13A2^−/−^ cells. (**D**) The viability of ATP13A2^−/−^ cells was significantly reduced upon exposure to ZnCl_2_ (112.5 µm), while no difference was observed in the control. Further to this, co-treatment with IBMX (100 µm) blocked Zn^2+^-mediated cytotoxicity in ATP13A2^−/−^ cells. Values in the graphs are represented as mean ± SD. CCCP, carbonyl cyanide 3-chlorophenylhydrazone; IBMX, 3-isobutyl-1-methylxanthine. ##*P* < 0.01 by Mann–Whitney *U* test and *P < 0.05 and **P < 0.01 by Kruskal–Wallis one-way ANOVA followed by *post hoc* Tukey's HSD multiple comparison test.

Dysfunctional mitochondria, when excessively damaged by toxic stimuli such as ROS to an extent beyond the cellular capacity to restore their normal function by complementation, undergo fragmentation before uptake by autophagosomes and delivery to lysosomes for degradation, the process known as mitophagy (40). Our observation of Zn^2+^-induced mitochondrial dysfunction prompted us to investigate the potential effect of increased Zn^2+^on mitochondrial morphology. We treated hONs cells with ZnCl_2_ and determined the mitochondrial reticular interconnectivity by calculating the mitochondrial form factor, of which low values indicate a more fragmented mitochondrial network and high values indicate a more cohesive reticulum (see Materials and Methods for details). When grown in media with a vehicle, there was no difference detected in the form factor between the cell lines (Fig. 7A and B). Whereas, upon the addition of CCCP to the media, dramatic changes in mitochondrial network morphology were observed in both the cell lines, with significantly lower form factors compared with the respective vehicle controls (*P* < 0.01 for both cell lines). Upon exposure to ZnCl_2_, ATP13A2^−/−^ cells displayed an average reduction of 29% in the form factor indicative of mitochondrial fragmentation when compared with the control (*P* < 0.01) and the vehicle-treated counterpart (*P* < 0.01). Conversely, the form factor for control cells under ZnCl_2_ treatment was similar to the vehicle control counterpart, revealing a Zn^2+^-specific effect on mitochondrial morphology in the absence of ATP13A2. Zn^2+^-induced mitochondrial fragmentation was completely blocked by co-treating the cells with a mitochondrial fusion promoter, 3-isobutyl-1-methylxanthine (IBMX) (*P* < 0.01). We also examined the effect of Zn^2+^-induced mitochondrial fragmentation on the cellular ATP production rate and cell viability (Fig. 7C and D). ZnCl_2_ significantly impaired ATP production in both cell lines, but to a greater extent in the ATP13A2^−/−^ cells (23.4 ± 1.1 for vehicle and 13.8 ± 0.4 for ZnCl_2_ treatment, *P* < 0.01) when compared with the control groups (24.9 ± 1.0 for vehicle and 21.0 ± 0.9 for ZnCl_2_ treatment, *P* < 0.05). The ATP production rate was significantly restored by co-treatment with ZnCl_2_ and IBMX in the ATP13A2^−/−^ cells (16.7 ± 0.5, *P* < 0.05 compared with ZnCl_2_ treatment). In addition, IBMX treatment reversed the Zn^2+^-induced reduction in cell viability for ATP13A2^−/−^ cells (*P* < 0.05, Fig. 7D). These findings indicate that Zn^2+^-induced mitochondrial fragmentation causes a reduction in ATP production that leads to cell death in ATP13A2^−/−^ cells.

## DISCUSSION

We demonstrate that ATP13A2 plays a crucial role in maintaining zinc homeostasis in a KRS-patient-derived cell model that lacks ATP13A2. The ATP13A2-deficient patient hONs cells showed abnormal zinc metabolism including low [Zn^2+^]_i_, altered expression of ZnTs/ZIPs and impaired sequestration of Zn^2+^ into ALP vesicles.

Several *in vitro* models have been used to manipulate ATP13A2 expression and show variation in cellular responses to Mn^2+^; Yeast devoid of YPK9, an orthologue of human ATP13A2, showed an increased sensitivity to Mn^2+^, while overexpression conferred resistance (16). In studies using mammalian cell models, Mn^2+^ at high concentrations (>1 mm) increased cell death and also induced expression of endogenous *ATP13A2* mRNA, while overexpression of wild-type ATP13A2, but not pathogenic variants, protected against its toxic effect (9, 10). Contrary to these findings, it has been demonstrated that overexpressed human ATP13A2 failed to protect cells against Mn^2+^ toxicity, raising questions over the biological relevance of the function of human ATP13A2 in manganese metabolism (41).

In this study, we have demonstrated abnormal zinc metabolism in the setting of ATP13A2 deficiency in KRS-patient-derived hONs cells. Increased sensitivity to the exogenous application of ZnCl_2_ and H_2_O_2_ (both of which increase [Zn^2+^]_i_ by direct uptake into cells for ZnCl_2_ and oxidant-induced release from zinc-binding proteins for H_2_O_2_), together with the protective effects of antioxidant treatment (NAC) and Zn^2+^ chelation (TPEN), underpin the pathophysiology of Zn^2+^ toxicity in ATP13A2-deficient cells (Fig. 1). Moreover, ATP13A2^−/−^ cells had a significantly lower [Zn^2+^]_i_ (Fig. 2) and altered mRNA expression of ZnTs/ZIPs (Fig. 3), indicating compensatory changes in ATP13A2^−/−^ cells and thus, providing strong support for zinc dyshomeostasis in the setting of ATP13A2 deficiency. By using hONs cells expressing mRFP-LC3 to investigate Zn^2+^ sequestration in the ALP vesicles (Fig. 4), we were able to show that fewer Zn^2+^ containing mRFP-LC3-positive vesicles were present in ATP13A2^−/−^ cells, suggesting impaired vesicular sequestration of Zn^2+^ and therefore a reduced capacity to buffer Zn^2+^. The fact that mRFP-LC3-positive vesicles accumulated in cells to a similar degree is confirmatory of a genuine difference in co-localization. Although not significant, the observed increase in the FluoZin-3 fluorescence intensity per vesicle in ATP13A2^−/−^ cells may reflect a protective response to buffer Zn^2+^ via other ZnTs in the setting of ATP13A2 deficiency.

Tsunemi *et al*. (accepted manuscript co-submitted to HMG: HMG-2013-W-00998.R1) also reported increased toxicity to Zn^2+^ with a lack of sensitivity to several biometals, including Mn^2+^ in KRS-patient-derived fibroblasts and *ATP13A2*-silenced primary neurons. These data, together with our findings, suggest that human ATP13A2 preferentially functions as a regulator for zinc rather than manganese, while ATP13A2 homologues from other species (e.g. yeast) likely have different substrate selectivities. Further investigations on the protein structure of ATP13A2 from various species and the amino acid residues involved in the interaction with substrates would be helpful in understanding the differences in species-specific cationic selectivity.

A number of ZnTs/ZIPs have been identified in the ALP vesicles, including lysosomes (e.g. ZnT2, ZnT4 and ZIP8, see Kambe *et al* (35) for a review), implicating the involvement of Zn^2+^ in lysosomal function. Although the molecule pumping Zn^2+^ in autophagosomes has not yet been identified, a recent study showed the existence of potential ZnTs in autophagosomes and the crucial role of Zn^2+^ in the normal function of autophagy (37). We showed the decreased capacity for sequestration of Zn^2+^ into the ALP vesicles and increased Zn^2+^ toxicity in ATP13A2^−/−^ cells, suggesting that ATP13A2 functions as a common Zn^2+^ regulator for the pathway to protect cells from the toxicity of excessive Zn^2+^. Such a protective function has also been observed for ZnT2, which accumulates Zn^2+^ into target cellular organelles and blocks Zn^2+^ toxicity (42). While our data indicate that ATP13A2 facilitates sequestration of Zn^2+^ into the ALP vesicles, it is not clear whether ATP13A2 is involved in the transportation of Zn^2+^ from the ALP vesicles to cytosol under physiological [Zn^2+^]_i_. The elevated level of *ZnT4* transcripts and the lack of *ZIP8* expression observed in our patient cells are suggestive of a bidirectional function for ATP13A2 due to the reported localization of these transporters in lysosomes/endosomes. Further studies measuring vesicular Zn^2+^ using radioactive ^65^Zn in the control and patient-derived cells under patho/physiological [Zn^2+^]_i_ are warranted to confirm the role of ATP13A2 in zinc transport.

Several studies have reported mitochondrial dysfunction in KRS-patient-derived fibroblasts and mammalian cell models (6, 28, 39). Consistent with these, we also observed mitochondrial dysfunction, as characterized by a reduction in ATP production and Δ*Ψ*_m_, in our ATP13A2^−/−^ cells (Figs 5 and 6). Our patient cells showed decreased levels of ROS production under normal growing conditions (Fig. 5C); a state which may be due to efficient ROS removal, as implicated by the increased mRNA expression levels of antioxidant proteins (Fig. 5D and below). In agreement with the suggested role of ROS as an effector of Zn^2+^-mediated toxicity (Fig. 1), exogenous Zn^2+^ increased H_2_O_2_ production in ATP13A2^−/−^ cells (Fig. 5C). The failure to detect a difference in mitochondrial O_2_^−^ production (data not shown) may alternatively be due to a short half-life of O_2_^−^ or the subtle difference in O_2_^−^ levels induced by ZnCl_2_ treatment. Our data are clearly in line with previous reports that have shown Zn^2+^ translocation into mitochondria via Zn^2+^ transporting uniporters (36) followed by an increase in ROS production (34), although the exact mechanisms involved in this process are still unclear.

Furthermore, we observed an oxidant-induced increase in [Zn^2+^]_i_ resulting in mitochondrial depolarization in ATP13A2^−/−^ cells, which was effectively prevented by Zn^2+^ chelation with TPEN (Fig. 6). Damaged and dysfunctional mitochondria that are incapable of carrying out their normal function undergo fragmentation via inhibition of mitochondrial fusion before degradation (40). Consistently, we have observed a more fragmented mitochondrial network in patient fibroblasts whose mitochondria were inherently dysfunctional (28). However, we detected no difference in hONs cells, which could be due to cell-specific differences between the cell lines. Nevertheless, exogenous Zn^2+^ administration was capable of inducing mitochondrial fragmentation in ATP13A2^−/−^ cells (Fig. 7). Increased ROS levels are known to induce mitochondrial damage, thereby activating the mitochondrial fission pathway and inhibiting the fusion pathway in order to segregate dysfunctional mitochondria from the healthy reticulum (43). Mitochondrial fragmentation induced by Zn^2+^ in our hONs cells was likely mediated by ROS production induced by Zn^2+^ (Fig. 5). The adverse effect of elevated [Zn^2+^]_i_ on mitochondrial morphology was blocked by IBMX treatment, resulting in an extensively interconnected mitochondrial network (Fig. 7). IBMX is known to induce accumulation of cAMP by inhibiting its degradation, in turn activating protein kinase A, which phosphorylates dynamin-related protein 1 (DRP1), an essential mitochondrial fission factor (44). Phosphorylation of DRP1 prevents it from interacting with the mitochondrial outer membrane, thereby impeding mitochondrial fission in favour of mitochondrial fusion. As well as its effect on mitochondrial interconnectivity, exogenous Zn^2+^ was also found to cause ATP depletion and cell death (Fig. 7). These data indicate mitochondria as a primary target of Zn^2+^ toxicity in the setting of ATP13A2 deficiency. Promotion of mitochondrial fusion through the introduction of IBMX was beneficial in protecting cells from the toxic effects of Zn^2+^, highlighting the role of mitochondrial fragmentation in Zn^2+^ toxicity. These findings indicate that abnormal mitochondrial function is closely linked to ATP13A2 deficiency-mediated zinc dyshomeostasis, strongly supporting the loss of ATP13A2 as the cause of mitochondrial dysfunction in our KRS-patient-derived cell line.

Two recent studies reported an increase in ROS production, mitochondrial membrane potential and mitochondrial fragmentation in ATP13A2-silenced cells (6, 39), seemingly contradicting our observations in ATP13A2^−/−^ cells grown under the basal conditions. These changes were most likely caused by the toxicity of transiently increased [Zn^2+^]_i_ due to uncompensated impairment in the cellular Zn^2+^ buffering system upon the acute loss of ATP13A2. In contrast, our patient cells inherently harbouring ATP13A2 deficiency have demonstrated compensatory changes (e.g. altered expression of ZnTs/ZIPs and antioxidant proteins) which result in lowered [Zn^2+^]_i_ and ROS production, allowing the cells to avoid possible damage by Zn^2+^-induced ROS production. Despite the beneficial effect on cell survival, low [Zn^2+^]_i_ may also have caused mitochondrial dysfunction in ATP13A2^−/−^ cells due to its adverse effect on mitochondrial function as shown in TPEN-mediated impairment of Δ*Ψ*_m_ and ATP production (45, 46).

**Figure 8.**
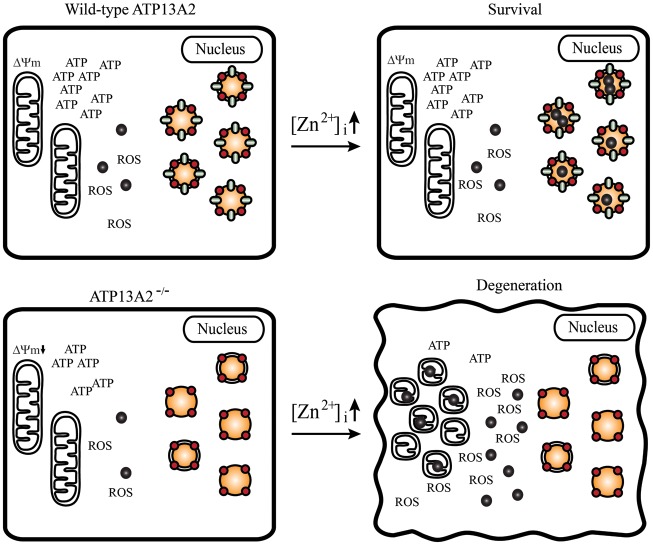
Schematic model of zinc dyshomeostasis and abnormal energy metabolism in ATP13A2 deficiency. Loss of ATP13A2 (green ellipse) results in a limited cellular buffering capacity for cytosolic Zn^2+^ due to the impairment in sequestration of Zn^2+^ (black circle) into LC3 (red circle) positive vesicles (single and double membraned organelles) associated with the ALP. The ensuing zinc dyshomeostasis results in mitochondrial dysfunction (lower Δ*Ψ*_m_ and ATP levels). When the [Zn^2+^]_i_ is elevated, cytosolic Zn^2+^ levels also increase due to inefficient sequestration by LC3-positive vesicles in the setting of ATP13A2 deficiency and instead induce the accumulation of Zn^2+^ in mitochondria, which increases ROS production. An elevated level of ROS in turn causes mitochondrial damage, worsening mitochondrial dysfunction that subsequently leads to reduced energy production, fragmentation of the mitochondrial network and cellular degeneration due to ATP depletion.

A schematic model summarizing the pathogenic mechanisms of how ATP13A2 deficiency likely causes zinc dyshomeostasis and mitochondrial dysfunction is illustrated in Figure 8; loss of ATP13A2 results in a limited cellular buffering capacity of cytosolic Zn^2+^ due to the impairment of Zn^2+^ sequestration by ALP vesicles and thus zinc dyshomeostasis, which in turn results in mitochondrial dysfunction. When the [Zn^2+^]_i_ is elevated, high levels of cytosolic Zn^2+^ induced by inefficient sequestration trigger mitochondria to increase their production of ROS, which in turn leads to mitochondrial damage when the level of accumulated ROS exceeds cellular antioxidizing capacity, causing aggravation of mitochondrial dysfunction and oxidative stress. Extensive dysfunction in mitochondria causes mitochondrial fragmentation, leading to ATP depletion and consequently cellular degeneration.

In this study, we show that human ATP13A2 is involved in Zn^2+^ transportation into the ALP vesicles and a loss of which results in zinc dyshomeostasis and abnormal energy metabolism. Our results indicate that human ATP13A2 is a common molecule associated with the mechanisms underlying zinc dyshomeostasis and mitochondrial dysfunction. The findings extend our current knowledge of the pathogenesis of PD, which may facilitate the development of a neuroprotective strategy to treat PD.

## MATERIALS AND METHODS

### Chemicals

All chemicals used here were purchased from Sigma (St Louis, MO, USA) unless stated otherwise.

### Cell culture

The protocols for establishment and culture of hONs cell lines have previously been described (3). hONs cells were subcultured to a maximum of 10 passages for all experiments. This study was approved by the Northern Sydney & Central Coast Health Human Research Ethics Committee.

### Lentivirus production and establishment of cell lines

V5-tagged wild-type ATP13A2 (V5ATP13A2) in pcDNA3-V5ATP13A2 (3) was subcloned into a pER4 lentiviral vector. Lentivirus for the expression of mRFP-LC3 (38) and V5ATP13A2 was produced using the Lenti-X Lentiviral Expression system (Clontech, Mountain View, CA, USA) and Lipofectamine 2000 (Invitrogen, Carlsbad, CA, USA) according to the manufacturer's instruction. The medium containing lentivirus was collected at 48 and 72 h post-transfection followed by concentration using the Lenti-X concentrator before measurement of viral titre.

hONs cells were transduced with one to two multiplicity of infection (MOI) lentivirus in the presence of 4 µg/ml polybrene for 24 h and used for subsequent experiments. Expression of target molecules in the cells was confirmed by western blotting according to the previously published protocol (3) or fluorescence microscopy. For generation of stable cell lines expressing mRFP-LC3, the cells were grown in culture media containing 1 µg/ml puromycin for selection.

### Neutral red uptake assay

hONs cells were plated at 5 × 10^4^ cells per well in a 24-well plate and grown to confluency. Following incubation in serum-free media for 16–24 h, the cells were exposed to different combinations of test chemicals for 24 h as indicated. For IBMX treatment, cells were pre-treated with 100 µm IBMX for 16 h before co-treatment with ZnCl_2_. The Neutral red uptake assay for cell viability was performed according to a protocol described elsewhere (33).

### Quantification of transcripts for ZnTs/ZIPs and antioxidant enzymes

hONs were plated at 2 × 10^5^ cells per well in a six-well plate and grown for 24 h. Total RNA was extracted using the RNeasy Mini kit (Qiagen, Germany) and 2 µg of total RNA was used to synthesize complementary DNA (cDNA) using the Superscript III first-strand synthesis kit for RT–PCR (Invitrogen) following DNase I treatment (Promega, Madison, WI). qRT–PCR was performed using the QuantiTect SYBR green PCR kit (Qiagen) and specific primers for the genes encoding human ZIP and ZnT families (KiCqStart SYBR Green Primers, Sigma) in a Rotorgene 6000 real-time PCR machine (Qiagen) according to the manufacturer's instructions. Primers were annealed at 60°C over 45 cycles. Primers used to amplify the genes encoding antioxidant proteins are listed in Supplementary Material, Table S1. At the end of each qRT–PCR run, melting curve analysis was performed to confirm specific target gene amplification.

### Imaging of intracellular free zinc ions

[Zn^2+^]_i_ was assessed using the Zn^2+^-specific fluorescent dye, FluoZin-3 AM (Invitrogen), by live cell imaging. hONs cells (3 × 10^4^) were plated in the inner chamber of a 35 mm µ-Dish (ibidi, Germany) and grown for 24 h. The cells were then stained with 5 µm FluoZin-3 AM for 1 h in a cell culture incubator. After washing with Hank's balanced salt solution (HBSS), the cells were incubated with either ethanol/distilled water, 0.75 mm H_2_O_2_ or 0.75 mm H_2_O_2_ supplemented with 1 µm TPEN for 30 min.

In order to assess Zn^2+^ levels in the ALP vesicles, hONs cells expressing mRFP-LC3 were grown in µ-Dishes, as mentioned above. On the day of assay, the cells were treated with 100 nm bafilomycin A1 for 4 h, the last hour of which was in co-treatment with 5 µm FluoZin-3 AM. After removing extraneous dye by washing with HBSS, the cells were incubated with 0.75 mm H_2_O_2_ for 30 min, followed by confocal microscopy.

Fluorescence was visualized using a Leica SP5 confocal microscope (Leica, Germany). In each experiment, the same parameters were applied to acquire images from all samples. Image J software (version 1.43 m, National Institutes of Health, Bethesda, MD, USA) was used to analyse the images to determine fluorescence intensity and the co-localization coefficient.

### Monitoring of mitochondrial membrane potential (Δ*Ψ*_m_)

hONs cells were seeded in a black 96-well plate at 1 × 10^4^ cells per well and grown for 24 h. For assessment of Δ*Ψ*_m_, the cells were incubated with either dimethyl sulfoxide (DMSO) or 25 µm CCCP for 4 h in serum-free media. After washing with HBSS, the cells were stained with 25 nm TMRM for 15 min in a cell culture incubator before measurement of fluorescence using a Victor 3 V1420 multilabel plate counter (Perkin Elmer, Waltham, MA, USA).

To determine the effect of elevated [Zn^2+^]_i_ on Δ*Ψ*_m_, hONs cells plated in a 35 mm µ-Dish were incubated with either 0.9 mm H_2_O_2_ or 0.9 mm H_2_O_2_ with 1 µm TPEN for 5 h in serum-free media. In the last hour, the cells were co-incubated with 500 nm JC-1 (Invitrogen). Fluorescence was visualized using a Leica SP5 confocal microscope (Leica) with constant parameters applied to acquire images from all samples. The area occupied by mitochondria in red fluorescence per cell was calculated in morphologically intact cells using Image J software (version 1.43 m).

### Measurement of ROS production

hONs cells were plated at 5 × 10^4^ cells per well in a black 96-well microplate and grown to confluency. The cells were then stained with 5 µm CM-H_2_DCFDA (H_2_O_2_ indicator, Invitrogen) or 5 µm MitoSox Red (O_2_^−^ indicator, Invitrogen) for 15 min at 37°C. After washing off extraneous dyes with HBSS, the cells were treated with increasing doses of ZnCl_2_ and the fluorescence from cells was immediately measured using a Victor 3 V1420 multilabel plate counter (Perkin Elmer) with measurements every 5 min for 30 min.

### Assessment of ATP production rate

ATP production rate was determined following the previously described protocol (47). Briefly, the cells were harvested by trypsinization before determining the total protein concentration using a BCA protein assay kit (Thermo Scientific, Rockford, IL, USA) according to the manufacturer's instructions. Cells were diluted in a cell suspension buffer [150 mm KCl, 25 mm Tris–HCl pH 7.6, 2 mm EDTA pH 7.4, 10 mm KPO_4_ pH 7.4, 0.1 mm MgCl_2_ and 0.1% (w/v) BSA] at 1 mg/ml total protein. ATP synthesis was induced by incubation of 250 μl of the cell suspension with 750 μl of substrate buffer (10 mm malate, 10 mm pyruvate, 1 mm ADP, 40 μg/ml digitonin and 0.15 mm adenosine pentaphosphate) for 10 min at 37 °C. Following this incubation, the reaction was stopped by the addition of 450 µl of boiling quenching buffer (100 mM Tris-HCl, 4 mM EDTA pH 7.75) into a 50 µl aliquot of the reaction mixture and subsequently incubated for 2 min. The resulting reaction mixture was further diluted 1:10 in quenching buffer, and the quantity of ATP was measured in an FB10 luminometer (Berthold Detection Systems, Germany) using the ATP bioluminescence assay kit (Roche Diagnostics, Switzerland), according to the manufacturer's instructions.

### Determination of mitochondrial interconnectivity

Mitochondrial network interconnectivity was assessed according to the previously described protocol (28). Briefly, hONs cells grown on coverslips were treated with either 100 µm ZnCl_2_ or 10 µm CCCP or 100 µm ZnCl_2_ and 100 µm IBMX for 24 h and then fixed in 4% (w/v) paraformaldehyde. For ZnCl_2_ and IBMX co-treatment, the cells were pre-treated with IBMX for 16 h before initiation of co-treatment. After permeabilization with 0.1% (v/v) Triton X-100, mitochondria were labelled with an anti-Grp75 antibody (Abcam, Cambridge, UK) and the Zenon immunolabelling kit (Invitrogen) according to the manufacturer's protocols. Fluorescence signals were assessed by confocal microscopy. Image J software (version 1.44) was used to measure the length of the mitochondrial perimeter (*P*_m_) and the area of mitochondrion (*A*_m_). Mitochondrial interconnectivity was determined by calculating the form factor (form factor = [*P*_m_^2^]/[4π*A*_m_]).

### Statistical analysis

All experiments were repeated three times in triplicate and the values are expressed as percentage change relative to vehicle-treated control groups unless otherwise stated in the text. All datasets were tested for normality using the Shapiro–Wilk test and analysed for statistical significance using SPSS (version 21, IBM, Armonk, NY, USA). A *P*-value of <0.05 was considered to be statistically significant.

## SUPPLEMENTARY MATERIAL

Supplementary Material is available at *HMG* online.

## FUNDING

This work was supported by the Australian National Health and Medical Research Council project grant (APP1010839 to C.M.S.) and the Australian Brain Foundation Parkinson's Disease Award (J.-S.P. and C.M.S.). Funding to pay the Open Access publication charges for this article was provided by the Australian Brain Foundation.

## Supplementary Material

Supplementary Data
