# Supplementary material for: Parkinson's disease-associated human ATP13A2 (PARK9) deficiency causes zinc dyshomeostasis and mitochondrial dysfunction
Source: Hum Mol Genet. 2014 Jan 7;23(11):2802–15. doi: 10.1093/hmg/ddt623 (PMC4014187; doi:10.1093/hmg/ddt623)
Supplement: Supplementary Data [file supp_23_11_2802__index.html]

Parkinson's disease-associated human ATP13A2 (PARK9) deficiency causes zinc dyshomeostasis and mitochondrial dysfunction — Parkinson's disease-associated human ATP13A2 (PARK9) deficiency causes zinc dyshomeostasis and mitochondrial dysfunction — Parkinson's disease-associated human ATP13A2 (PARK9) deficiency causes zinc dyshomeostasis and mitochondrial dysfunction — Supplementary Data 

# Parkinson's disease-associated human ATP13A2 (PARK9) deficiency causes zinc dyshomeostasis and mitochondrial dysfunction

## Supplementary Data

Supplementary Data

**Files in this Data Supplement:**

- Supplementary Data - Docx file
- Supplementary Figure 1 - tif file
- Supplementary Figure 2 - tif file
- Supplementary Figure 3 - tif file
- Supplementary Figure 4 - tif file
- Supplementary Table 1 - tif file
