# Supplementary material for: Parkinson's disease-associated human ATP13A2 (PARK9) deficiency causes zinc dyshomeostasis and mitochondrial dysfunction
Source: Hum Mol Genet. 2014 Jan 7;23(11):2802–15. doi: 10.1093/hmg/ddt623 (PMC4014187; doi:10.1093/hmg/ddt623)
Supplement: Supplementary Data [file supp_ddt623_ddt623supp.docx]

**Materials and Methods**

**Cell viability/cytotoxicity test**

We measured the activity of lactate dehydrogenase that had been released into the culture media by dead cells using the CytoTox non-radioactive cytotoxicity assay (Madison, WI), according to the manufacturer’s instructions.

Trypan blue exclusion assay to measure cell viability was performed by staining cells with Trypan blue solution (0.02% as final concentration) for 5 min at room temperature and immediately counting Trypan blue-negative viable cells using a Countess Automated Cell Counter (Invitrogen, Carlsbad, CA) according to the manufacturer’s instruction.

**Flow cytometry**

hONs were seeded at ~70% confluency and grown for 24 hours. After washing with warm PBS, the cells were incubated with 10µM Zinpyr-1 for 30min in a cell culture incubator. Cells were collected by trypsinisation and analysed by flow cytometry, after washing off unincorporated dyes. The fluorescence intensity of each cell was measured using a FACSCalibur flow cytometer (BD, San Jose, CA). For measurement of mitochondrial mass, the cells were collected by trypsinisation and incubated with 100nM MitoTracker Green FM (Invitrogen, Carlsbad, CA) for 30 min at 37 °C. The cells were then washed with warm PBS and analysed using flow cytometry.

**Supplementary figure legends**

**Supplementary figure S1. Zn^2+^ induced ROS-mediated cytotoxicity in ATP13A2^-/-^ hONs cells.** Human olfactory neurosphere (hONs) cells were tested for zinc sensitivity using lactate dehydrogenase (LDH) activity released into the media (A-D) or the Trypan blue exclusion assay (E-G). (A) Increasing doses of ZnCl_2_ (0, 100, 112.5 and 125 µM) significantly elevated the LDH activity in the media of ATP13A2^-/-^ cells (grey bars) in a dose-response manner, while the control displayed no changes (white bars). (B) The LDH release in the media induced by 125 µM ZnCl_2_ was blocked by 1 mM NAC, an antioxidant agent. (C) H_2_O_2_ treatment (0, 0.85, 0.9 and 1 mM) increased cytotoxicity significantly and dose-dependently in ATP13A2^-/-^ cells. (D) Co-treatment of 1 mM H_2_O_2_ and 1 µM TPEN blocked the cytotoxic effect of H_2_O_2_ in ATP13A12^-/-^ cells. (E) Increasing doses of ZnCl_2_ (0, 100, 112.5 and 125 µM) significantly reduced Trypan blue-negative viable ATP13A2^-/-^ cells in a dose-response manner, while the control displayed toxicity at the highest dose used. (F) H_2_O_2_ treatment (0, 0.85, 0.9 and 1 mM) significantly decreased viability of ATP13A2^-/-^ cells in a dose-dependent manner. (G) The toxic effect of ZnCl_2_ and H_2_O_2_ on ATP13A2^-/-^ cells was blocked by co-treatment with NAC and TPEN, respectively. Values in the graphs are represented as mean ± SD. NAC; N-acetyl-cysteine, TPEN; N,N,N',N'-Tetrakis(2-pyridylmethyl)ethylenediamine. *; p<0.05 and **; p<0.01 by Kruskal-Wallis one-way ANOVA followed by *post hoc* Tukey’s HSD multiple comparison test.

**Supplementary figure S2. ATP13A2^-/-^ cells display significantly lower levels of [Zn^2+^]_i_ in flow cytometry.** Human olfactory neurosphere (hONs) cells grown under normal conditions were stained with Zinpyr-1 and analysed using flow cytometry. The Zinpyr-1 signals from ATP13A2^-/-^ cells (grey bar) were significantly lower compared to the control (white bar). The experiment was performed in triplicate and >10,000 cells were analysed for each sample. Values in the graphs are represented as mean ± SD. *; p<0.05 in two-tailed Student’s *t*-test.

**Supplementary figure S3. Mitochondrial mass analysis of human olfactory neurosphere cell lines.** MitoTracker green was used to determine mitochondrial mass in the human olfactory neurosphere (hONs) cell lines. Analysis using flow cytometry found comparable levels of MitoTracker green signals in the control (white bar) and ATP13A2^-/-^ cells (grey bar). The experiment was performed in triplicate. Values in the graphs are represented as mean ± SD.

**Supplementary figure S4.** **Lack of cytotoxicity to high doses of ZnCl_2_ over a short exposure.** Cell viability was tested in human olfactory neurosphere (hONs) cells using the Trypan blue exclusion assay after exposure to 1000 µM ZnCl_2_ for 30 min. Both control (white bar) and ATP13A2^-/-^ (grey bar) cells did not show a reduction in cell viability under the given treatment condition. The experiment was performed in triplicate. Values in the graphs are represented as mean ± SD.
