# Supplementary figures and images for: Parkinson's disease-associated human ATP13A2 (PARK9) deficiency causes zinc dyshomeostasis and mitochondrial dysfunction
Source: Hum Mol Genet. 2014 Jan 7;23(11):2802–15. doi: 10.1093/hmg/ddt623 (PMC4014187; doi:10.1093/hmg/ddt623)

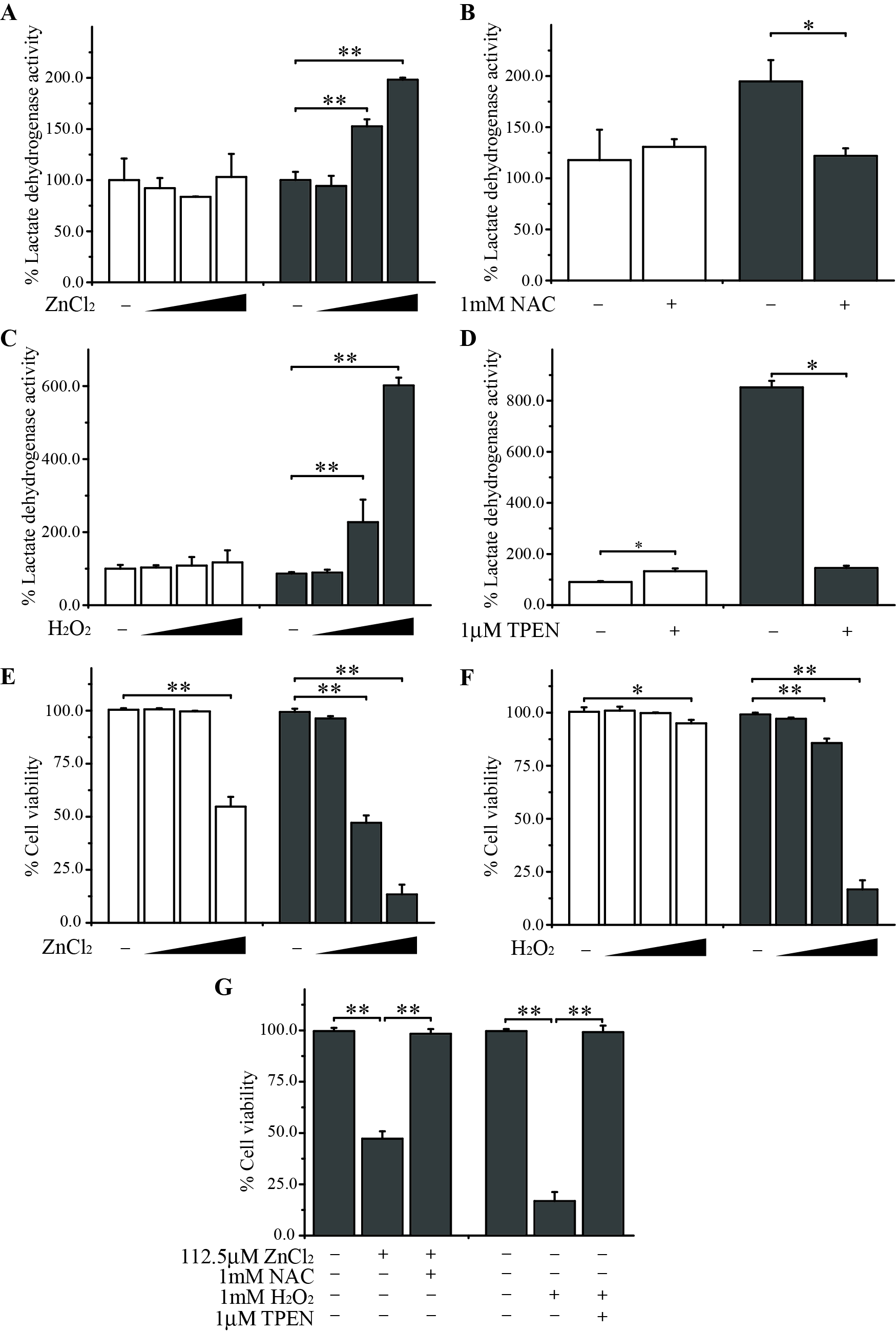

Supplement: Supplementary Data [file supp_ddt623_ddt623supp_fig1.tif]

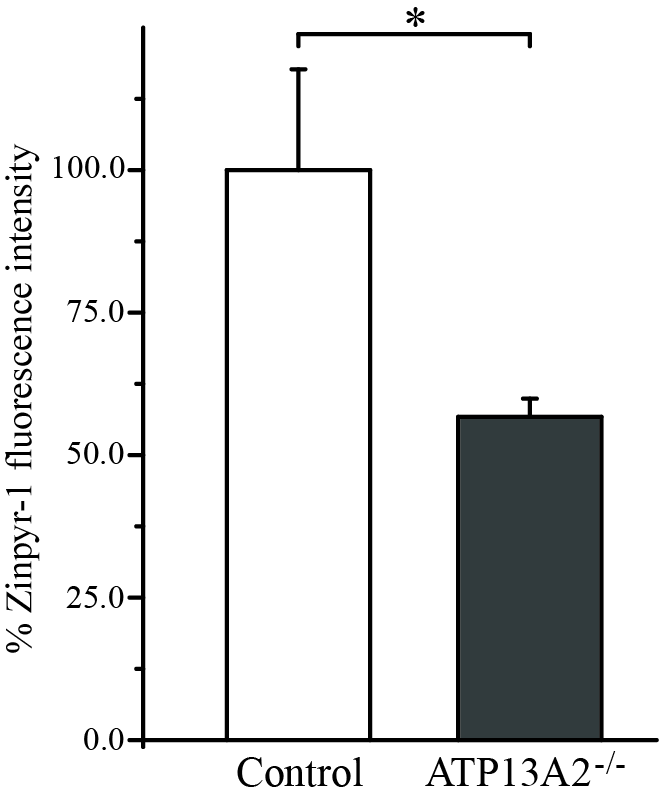

Supplement: Supplementary Data [file supp_ddt623_ddt623supp_fig2.tif]

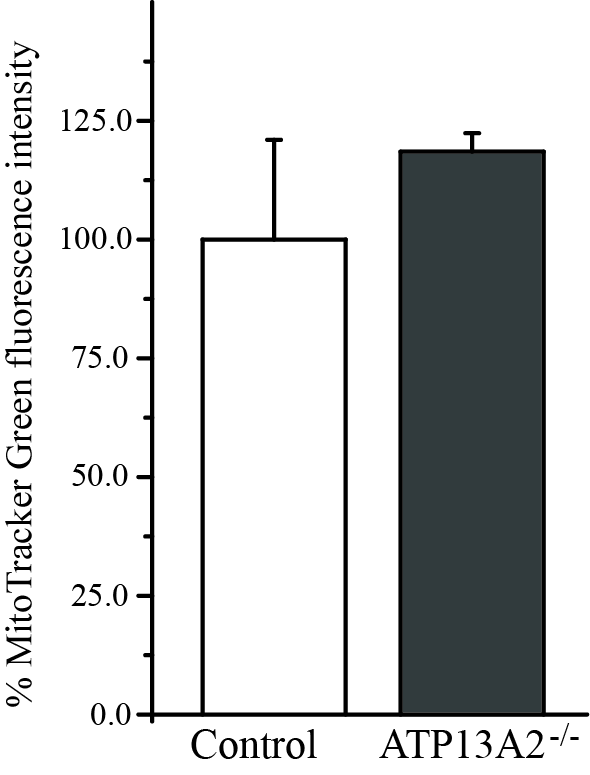

Supplement: Supplementary Data [file supp_ddt623_ddt623supp_fig3.tif]

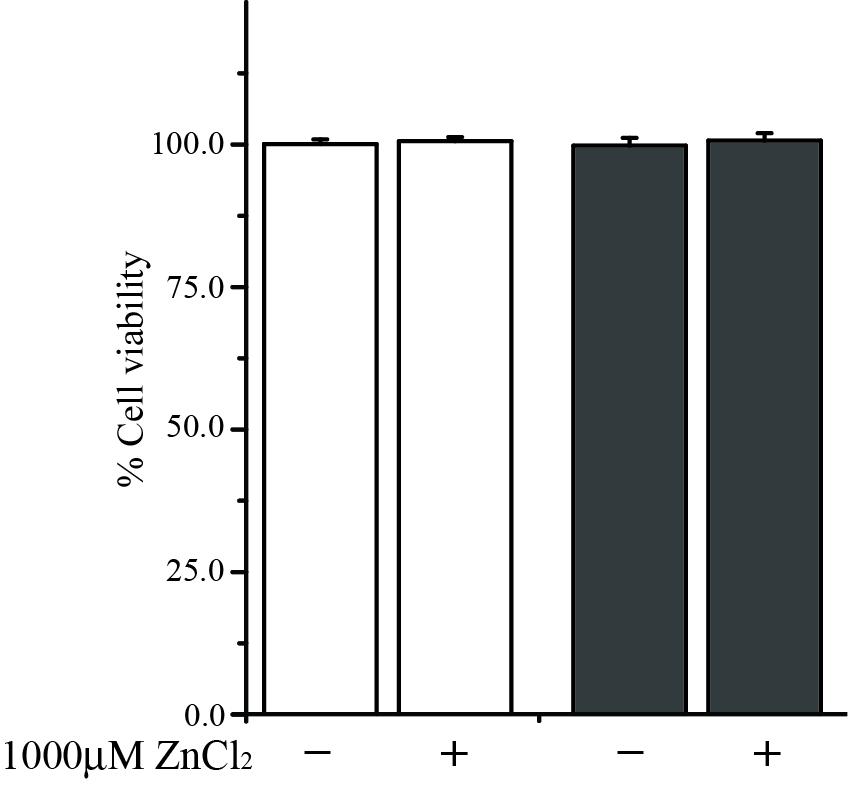

Supplement: Supplementary Data [file supp_ddt623_ddt623supp_fig4.tif]

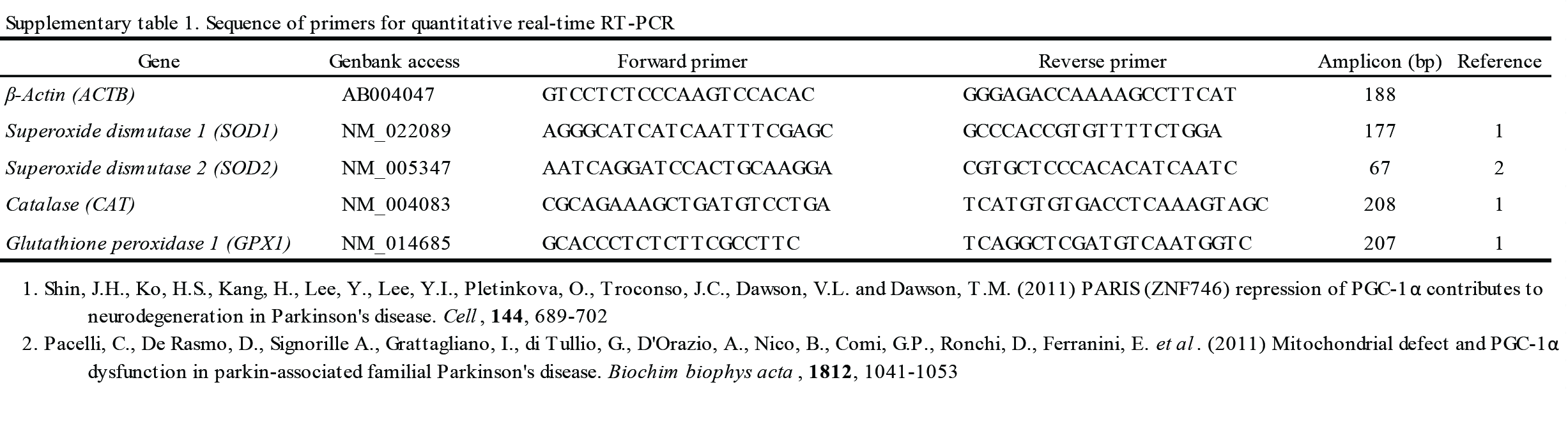

Supplement: Supplementary Data [file supp_ddt623_ddt623supp_table1.tif]
